# Supervised machine learning models for depression sentiment analysis

**DOI:** 10.3389/frai.2023.1230649

**Published:** 2023-07-19

**Authors:** Ibidun Christiana Obagbuwa, Samantha Danster, Onil Colin Chibaya

**Affiliations:** Department of Computer Science and Information Technology, School of Natural and Applied Sciences, Sol Plaatje University, Kimberley, South Africa

**Keywords:** Twitter, depression, sentiment analysis, text pre-processing, machine learning techniques, social media, natural language processing, mental health

## Abstract

**Introduction:**

Globally, the prevalence of mental health problems, especially depression, is at an all-time high. The objective of this study is to utilize machine learning models and sentiment analysis techniques to predict the level of depression earlier in social media users' posts.

**Methods:**

The datasets used in this research were obtained from Twitter posts. Four machine learning models, namely extreme gradient boost (XGB) Classifier, Random Forest, Logistic Regression, and support vector machine (SVM), were employed for the prediction task.

**Results:**

The SVM and Logistic Regression models yielded the most accurate results when applied to the provided datasets. However, the Logistic Regression model exhibited a slightly higher level of accuracy compared to SVM. Importantly, the logistic regression model demonstrated the advantage of requiring less execution time.

**Discussion:**

The findings of this study highlight the potential of utilizing machine learning models and sentiment analysis techniques for early detection of depression in social media users. The effectiveness of SVM and Logistic Regression models, with Logistic Regression being more efficient in terms of execution time, suggests their suitability for practical implementation in real-world scenarios.

## 1. Introduction

It is critical to understand people's emotions and daily online activities. Many researchers are interested in this topic because depression is a major cause of mental health problems that manifest themselves through social media posts. Twitter is one of the most popular social media platforms, with many people using it for person-to-person communication and sharing common interests based on their perspectives on real-life events (Ricard et al., 2018; Sood et al., 2018). Sentiment analysis can be used to monitor various social media sites in real-time. In short, Twitter will be used to classify the sentiment polarity of a tweet as positive, negative, or neutral (Babu and Kanaga, 2022), because tweets and written text appear to be incomplete and unstructured in nature.

This study uses a machine learning approach to create models that will help identify depressed social media users or persons earlier and help before it is too late. To accomplish this, data pre-processing, which included data cleaning, tokenization, stop words removal, stemming, lemmatization, bigram creation, sentiment classification, duplicate removal, and URL and number removal to improve tweet content was carried out. Machine learning classifiers: XGB Classifier, Random Forest, Logistic Regression, and support vector machine was used to build depression sentiment models, and the four classifiers performed excellently on the datasets.

The remainder of the paper is organized as follows: Section 2 introduces existing relevant work in the literature. Section 3 explains how the model is developed methodologically and practically. Section 4 displays the outcomes of our proposed approach's performance. Section 5 highlights the main points of the research. Finally, Section 6 presents the main conclusions of this paper.

## 2. Literature review

### 2.1. Depression

**Figure 1:**
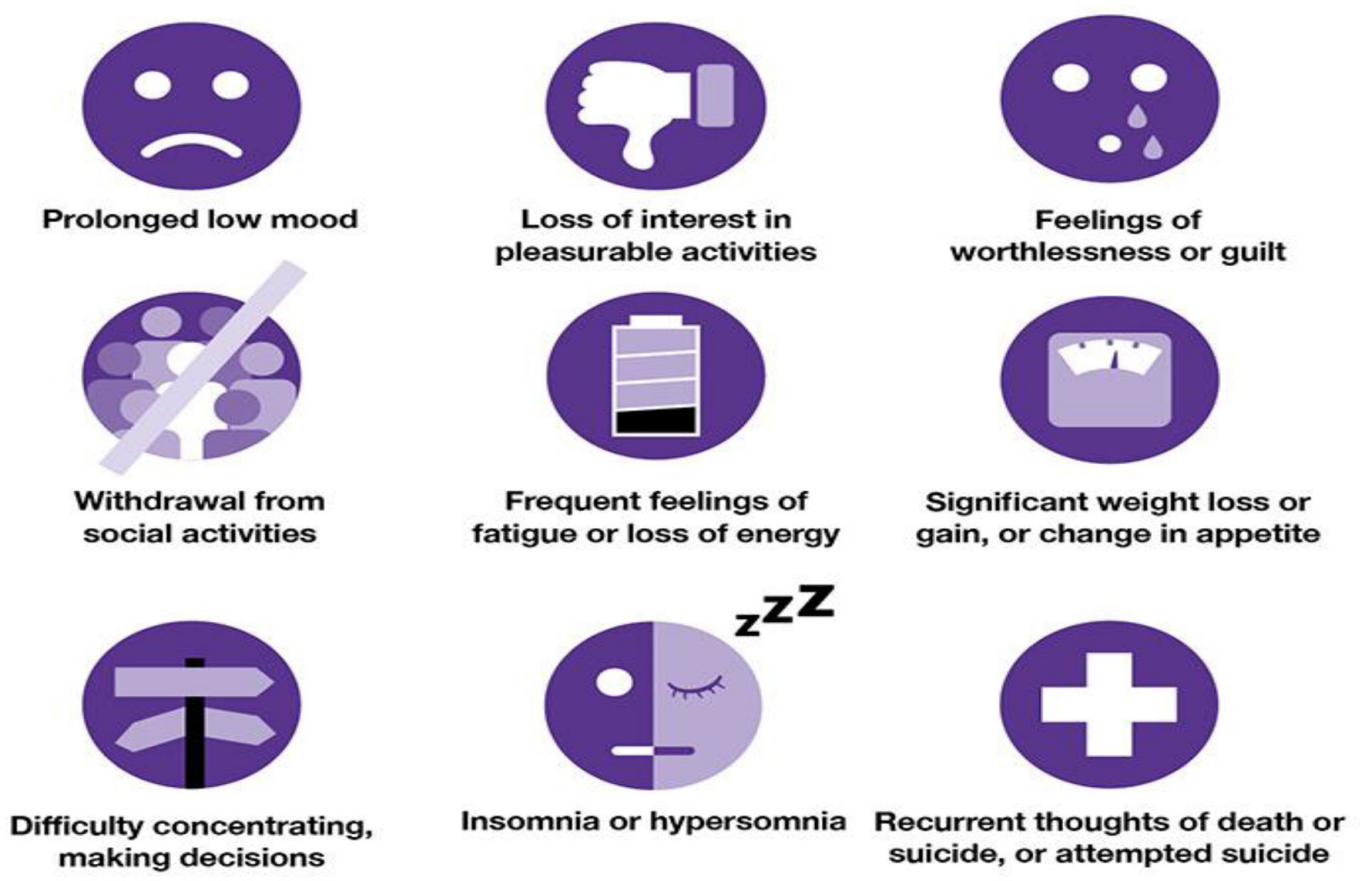
Depression symptoms (Source: The University of Queensland, 2022).

Depression is a major public health issue that affects people psychologically all over the world. It is defined as a collection of mixed impairment symptoms and disturbance in one's cognition and behavior (Orabi et al., 2018). During the 2019–2021 COVID era, there was a rapid increase in mental health issues and suicidal cases (Zulfiker et al., 2021). The World Health Organization states that more than 300 million people worldwide suffer from depression, prompting many researchers to focus on this topic (Priya et al., 2020). Chronic diseases can also be caused by depression. Figure 1 illustrates the symptoms of depression in a detailed manner.

Depression affects men and women differently, in such a way that women experience depression more severely than men (Seney et al., 2018). Because women are more likely to have anxiety disorders. Women are more involved in social gatherings, which exposes them to intimacy and emotional disclosure. Mental health issues can also be exacerbated by modern lifestyles influenced by social media and the pressure to live up to a certain standard that requires the approval of others (Kumar et al., 2020). Furthermore, studies revealed that younger individuals are disproportionately affected by mental health issues such as anxiety, depression, and obsessive-compulsive behavior, which has led many to commit suicide or consider it (Orsolini et al., 2021).

### 2.2. Social media posts linked with mental health issues on social media platforms

People use social media to share and communicate their ideas, and emotional states of being across many social platforms. Imagery is also a popular method of self-expression on social media platforms such as instagram (Mun and Kim, 2021). More evidence of depression can be found in Instagram photos (Smith and Anderson, 2018). As a result, this method effectively elicits deeper psychological consciousness by allowing emotional expressions that cannot be expressed in writing.

Another popular way to deal with mental health issues is through expressive writing or texting. Users of these social media platforms tend to document and narrate their lives through these platforms making it easy for us to understand their personal lives (Orabi et al., 2018). Twitter and Facebook are also popular social media platforms and important for person-to-person communication where people share their perspectives on real-life events (Ricard et al., 2018; Sood et al., 2018). Thus, depression can be identified through word sentiment analysis (Seabrook et al., 2018). According to Ricard et al. (2018), community-generated content responses can be used to identify levels of depression in people who have similar user-generated content. In retrospect, social media can also be beneficial to people's mental health.

### 2.3. Different methods of mining social media data

Before this paper can go into what social media mining entails, it is necessary to comprehend what this topic stems from. Social media mining stems from data mining (Babu and Kanaga, 2022), which could also be considered as a stem of machine learning in various fields (Babu and Kanaga, 2022). Data mining can be considered the process of finding useful sets of data from larger pools of accessible data (Jagadishwari et al., 2021; Babu and Kanaga, 2022). In some studies, this is considered an automated process of uncovering knowledge, relationships, and patterns in interrelated sets of data (Babu and Kanaga, 2022). Data mining techniques are used in the process of social media mining for several reasons and several methods could be implemented in this process.

The growth and impact of social media have grown rapidly over the past few years. With this growth, more and more data has become available for both private and commercial use through these platforms. Noticeably, we are all constantly interacting with each other through these social media platforms, and one could say, our whole lives are now documented through this data. From whom we talk to, whom we know, and what we like or dislike, this information is accessible to almost anyone through these platforms, with this, the analysis of people or their social cues can be done through this data. Social media mining can be considered a process of gathering interrelated data from social media platforms to identify patterns and relationships in the data (Babu and Kanaga, 2022). There are several forms in which this data could be found. Text, image, and voice are three common forms of data that are mined during the social media mining process (Jagadishwari et al., 2021).

#### 2.3.1. Text mining

**Figure 2:**
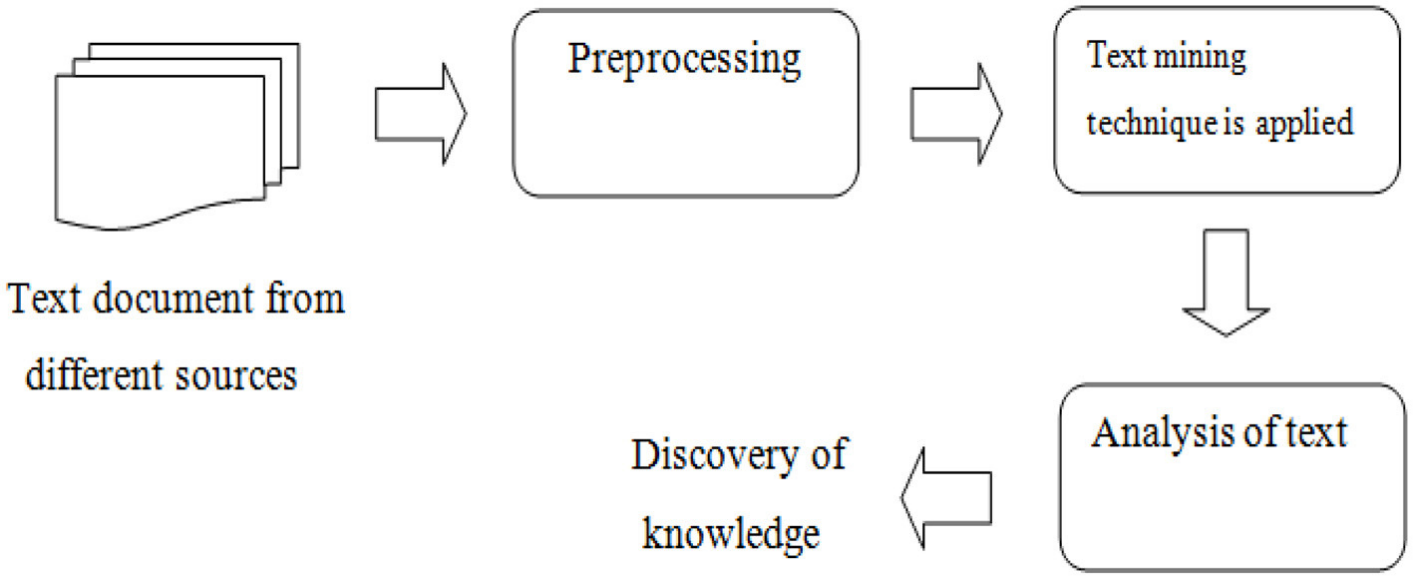
Text mining process (Source: Gaikwad et al., 2014).

This is the process of identifying relationships and patterns from large amounts of textual data to discover new knowledge or generate an understanding of some sort (Gaikwad et al., 2014). This process makes use of data mining algorithms and techniques such as classification, clustering, and association rules to discover new information and relationships in textual sources (Gaikwad et al., 2014; Babu and Kanaga, 2022). Several methods and techniques have been developed to solve the text mining problem according to the user's requirements, some of these methods include the term-based method (TBM) and the phrase-based method (PBM). The term-based method searches large amounts of text looking for similar terms, in this context term refers to text with a related language or logic (Gaikwad et al., 2014). The term-based method has the advantage of efficient computational performance and mature theories for term weighing. However, this method suffers from the problems of polysemy and synonymy (Gaikwad et al., 2014). Where polysemy refers to words with multiple meanings and synonymy refers to words with similar meanings. In the phrase-based method, the text is analyzed in phrases as these are less ambiguous and more discriminative in comparison to terms (Gaikwad et al., 2014). However, these phrase-based methods perform relatively poorly in comparison to a term-based method, and this could be because how phrases have inferior statistical properties in comparison to terms, these phrases also have a low occurrence frequency and large numbers of noise and redundant phrases are located among them (Gaikwad et al., 2014). Figure 2 depicts the process of text mining.

#### 2.3.2. Image mining

**Figure 3:**
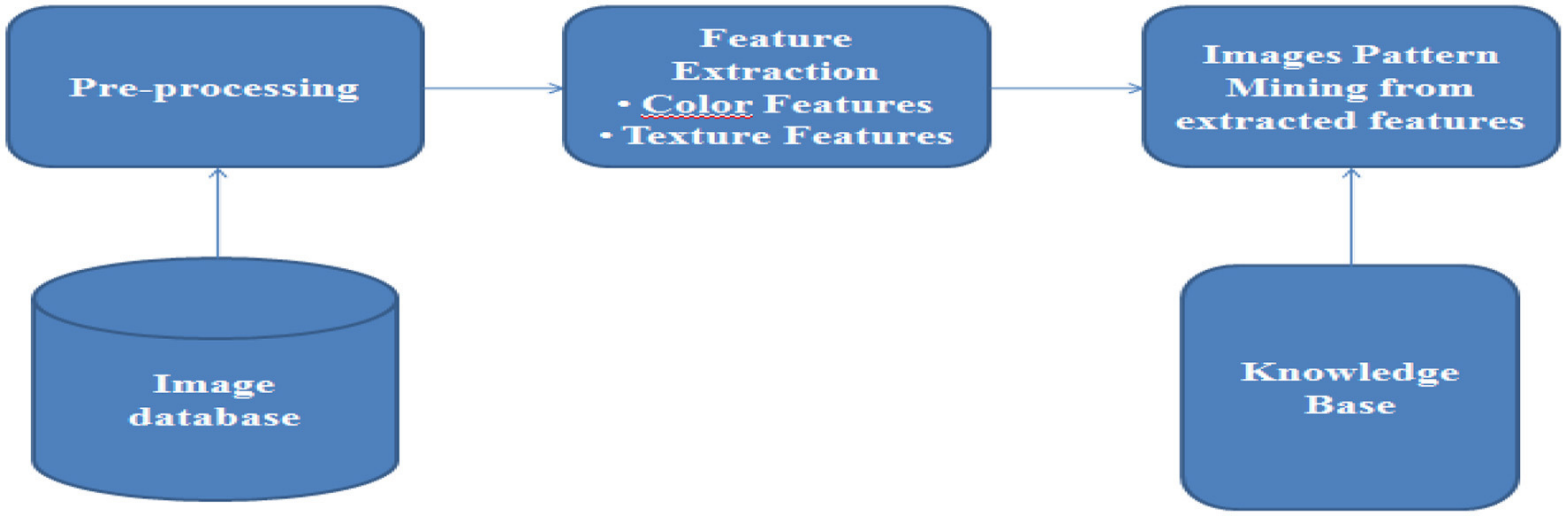
The image mining process (Source: Shukla and Vala, 2016).

Image mining is a subset of multimedia mining, which is used to extract informative and interesting graphical data (Shukla and Vala, 2016). Image mining, unlike computer vision and image processing techniques, focuses on extracting patterns from large collections of images (Shukla and Vala, 2016). Computer vision and image processing use a single image to understand or extract specific features (Shukla and Vala, 2016). Some of the common techniques centered around image mining include object recognition, image retrieval, image indexing, classification, clustering, and accusation rule mining (Shukla and Vala, 2016). The image mining process is shown in Figure 3 below.

#### 2.3.3. Voice mining

Voice mining is another subset of multimedia mining (Shukla and Vala, 2016). The use of voice has become very popular on social media platforms as these vocal messages contain emotional information and this emotional information has become a new topic in data mining and social media analytics (Babu and Kanaga, 2022). While it is not as popular as text mining or image mining now, there is clear growth, and this may become a major subset of the data mining and social media analytics sector.

For this study, Text mining techniques were utilized, as well as data mining algorithms and techniques to detect the rate of depression found in posts made by individuals on the Twitter social media application. Some of the most popular text mining methods are term-based mining (TBM) and phrase-based mining (PBM). Making use of single words to identify depression would not work because the word could mean different things depending on how it is used but considering how people generally use slang when posting on social media and all that, the models would have to adapt to this slang in order to make sense of the sentences. As a result, crucial clues regarding the sentences the word is used in or the context in which it is used would have been overlooked if a single word were to be identified as a trigger for depression.

### 2.4. Sentiment analysis for depression prediction

This is a growing topic that is used to understand people's sentiments about their everyday lives. This can be defined as a classification of text blocks, traditionally as either neutral, negative, or positive (Babu and Kanaga, 2022). However, this does not only depend on the polarity of the text, but the emotions associated with it, be it happy, sad angry, etc. Many studies have been conducted and commonly, various Natural Language Processing algorithms are used (Babu and Kanaga, 2022). Studies also show that Binary and Ternary classification techniques are regularly used, with multi-class classification providing more accurate results (Babu and Kanaga, 2022). Multi-class classification divides the data into multiple sub-classes and then works on these sub-classes separately based on the class's polarities (Gaikwad et al., 2014; Babu and Kanaga, 2022). Deep learning techniques are also used for the classification process (Gaikwad et al., 2014; Babu and Kanaga, 2022). However, the two common sentiment analysis techniques are rule-based sentiment analysis and machine learning-based sentiment analysis.

Rule-based sentiment analysis makes use of rules and word collections labeled by polarity to identify the opinion or context of the text (Babu and Kanaga, 2022). In this technique, sentiment value is made up of a combination of attributes to understand sarcasm, negation, or dependent clauses (Babu and Kanaga, 2022).

Machine learning-based sentiment analysis is focused on training a machine learning model using a sentiment-labeled training set (AlSagri and Ykhlef, 2020; Babu and Kanaga, 2022), to train the model to understand the polarity of words given in a certain order.

**Figure 4:**
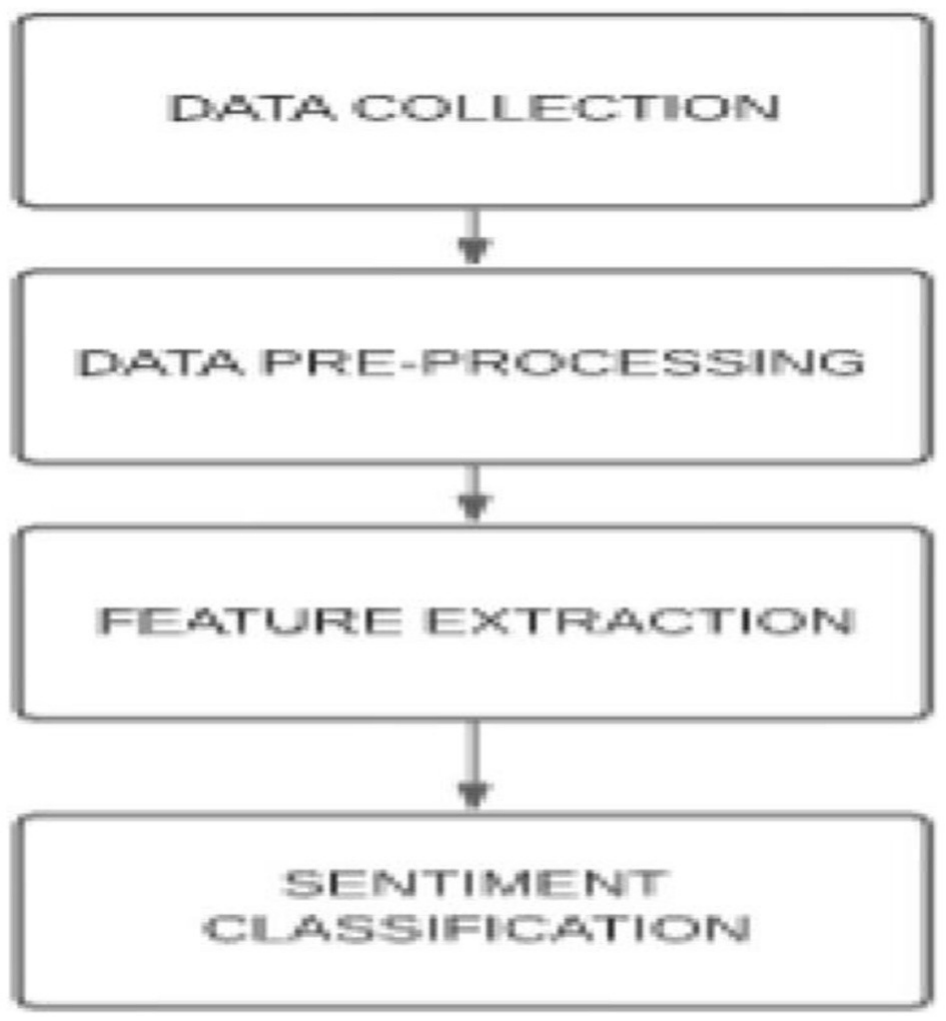
Sentiment analysis process (Source: Babu and Kanaga, 2022).

Sentiment analysis can be broken down into four major processes, namely, (1) data collection, (2) text preparation (data preprocessing), (3) sentiment detection (feature extraction), and (4) sentiment classification and presentation as output (Babu and Kanaga, 2022). The data collection process is made to allow for the relevant data to be collected, in this case, from various social media platforms. In a paper by Samsari et al. (2022) this data collection process consisted of a dataset made up of tweets collected during the COVID pandemic. Similarly in an article by Lui (2020), the data used in the study was collected from the Facebook and Twitter social media platforms. AlSagri and Ykhlef (2020), also collected data from tweets, and like most studies that made use of the text mining method, emoticons and emojis were removed as part of the preprocessing process (Babu and Kanaga, 2022). The data preparation process is used to clean the data by removing unrelated data and removing noise and words with no analytical significance (Babu and Kanaga, 2022). The third is the sentiment detection process, here the text is analyzed to extract opinions, reviews, and feedback while removing any text related to facts or popular knowledge (Babu and Kanaga, 2022). Thereafter, the classification and presentation process are conducted (Babu and Kanaga, 2022). Samsari et al. (2022) made use of the Naïve Bayes classifier to classify the data as either positive, negative, or neutral. Jagadishwari et al. (2021) made use of the Linear regression model as well as the Support Vector Machine (SMV) model, and these two models generated similar results. Ranganathan and Tzacheva (2019) used the Support Vector Machine LibLinear in an article titled Emotion Mining in Social Media Data. Tiwari et al. (2021) analyzed the performance of five different classification models and the most accurate results were generated by the Decision Tree classifier. The sentiment analysis process is shown in Figure 4 below.

These studies suggest that the two most common sentiment analysis methods used are the Rule-based method and the Machine learning-based method (AlSagri and Ykhlef, 2020). Regardless of the method used, there are four major processes in the sentiment analysis life cycle as mentioned, (1) data collection, (2) text preparation (data preprocessing), (3) sentiment detection (feature extraction), and finally (4) sentiment classification and preparation for output. Naïve Bayes, Decision Trees, SVM's, and Linear regression are some of the most common classification models used. However, studies suggest that Decision Trees have shown more accurate results when trained correctly (AlSagri and Ykhlef, 2020).

## 3. Methodology

### 3.1. Data collection and preparation

Four separate Twitter datasets were collected from Kaggle to narrow it down to three columns namely as one dataset: Tweet texts.

(i) Target (0, 1, 2)
(ii) The sentiment (positive, negative, and neutral)

**Figure 5:**
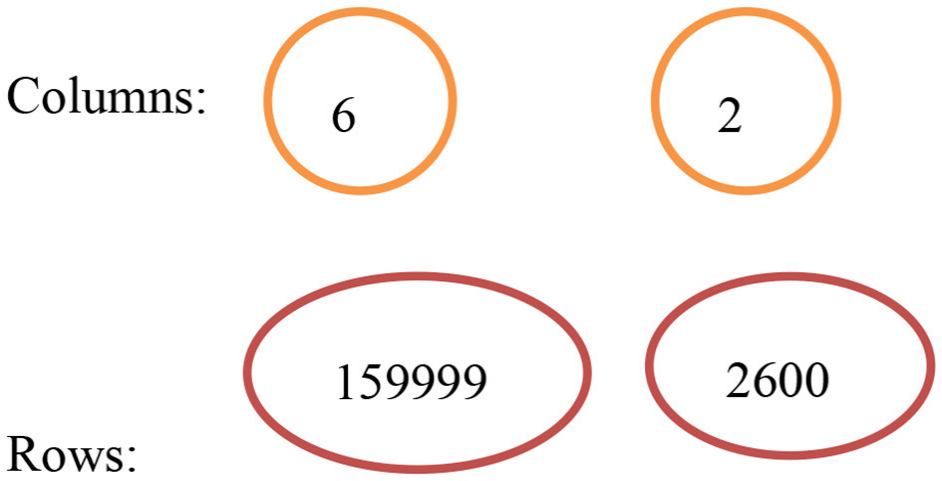
Before and after pre-processing.

Figure 5 is an illustration of the before and after pre-processing of the datasets merged as one dataset.

#### 3.1.1. Pre-processing

This process improves the quality of the dataset based on the tweets of the users. There are four datasets retrieved from the Kaggle website on depression. The datasets were cleaned the removing unnecessary features and merging the four datasets as one. For the text (Tweets) pre-processing, Natural Language Toolkit (NLTK), an open-source Python library for natural language processing techniques was employed to perform the following tasks:

i. Tokenization—Users' tweets are divided into several tokens, making stemming and word removal easier.
ii. Removal of Stop Words—Eliminating stop words such as “on,” “at,” and “the” to improve algorithm processing time.
iii. Stemming—Using stemming to identify the root of words in user tweets.
iv. Lemmatization—The “text normalization technique” will be used to bring tweets or words to their dictionary form. This process is like stemming, but the root words have meaning.
v. Creation of bigrams/trigrams—A bigram is two consecutive words in a sentence, while a trigram is three consecutive words in a sentence.

Furthermore, the tweets were classified as negative, positive, or neutral. Resulting in using negative reviews in relation to depression because texts or tweets about depression are perceived as negative. Duplicates were removed, sample description was carried out to determine how many ids and tweets are unique. Stop words were removed from the dataset, and special characters and links were replaced with blank spaces. URL links were removed from the corpus to improve tweet content. Numbers were removed because they are not useful for measuring sentiments. In addition, all the text was changed to lowercase.

#### 3.1.2. Appling target value to the different sentiments

Positive Sentiment: Target = 0

Negative Sentiment: Target = 1

Neutral Sentiment: Target = 2

#### 3.1.3. Baseline and evaluation

Two classification methods were used in this study. The first was a numerical classifier in which tweets were classified in a range of one to four, then the second was a three-way classifier which classified the tweets according to their polarity as either, negative, positive, or neutral. The numerical classifier was performed on all the datasets in order to generate a common target value. However, after the pre-processing stage, a single dataset containing a pre-existing sentiment column was used. The standard C-Method was then used in this research as a starting point technique and applied all six pre-processing methods, including removing URLs, removing stop words, removing numbers, reverting words that contain repeated letters to their original form, replacing negative mentions, and expanding acronyms to the original word. The accuracy and computational time are used to measure the overall classification process while the text pre-processing is measured by the loss or gain of accuracy.

#### 3.1.4. Sentiment visualization

To determine the most prevalent words, this study used word clouds in our dataset according to each sentiment (positive, negative, and neutral). Word clouds visualize the most frequent words in large sizes and the less frequent words in smaller sizes.

**Figure 6:**
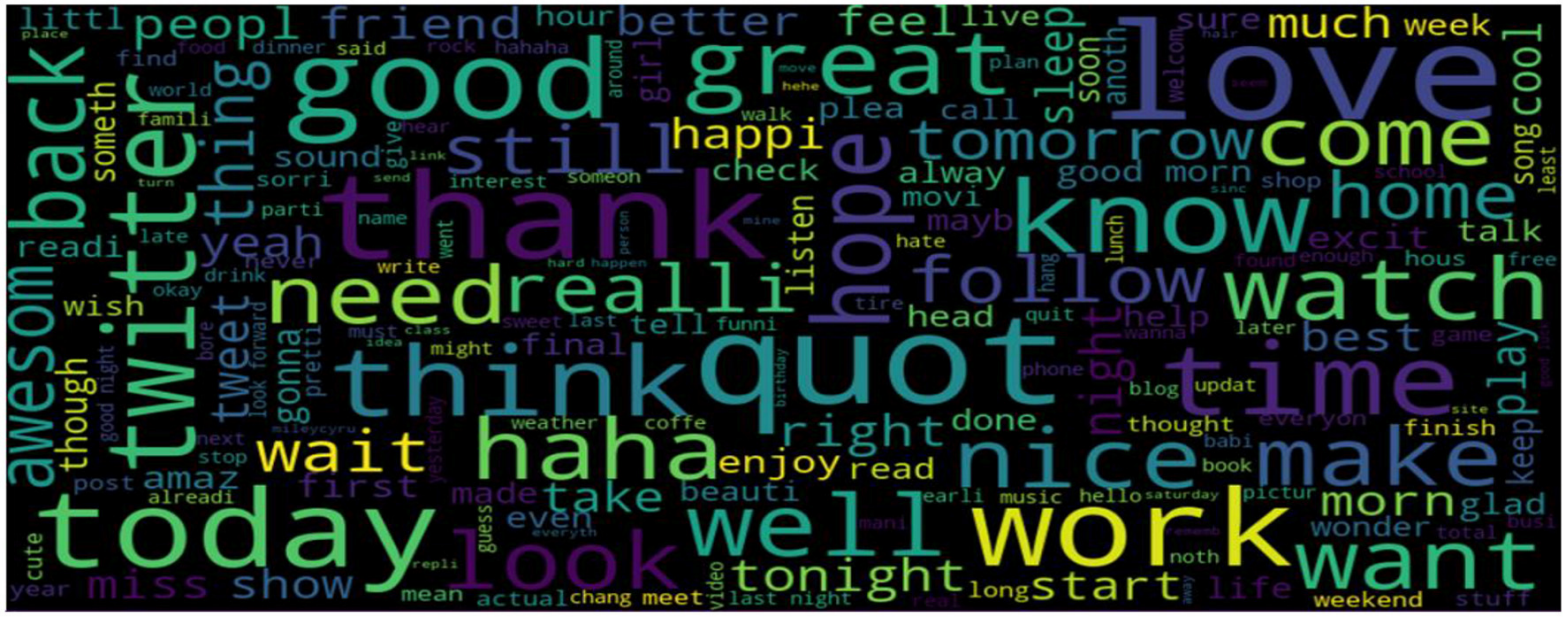
The most common words in the entire dataset.

**Figure 7:**
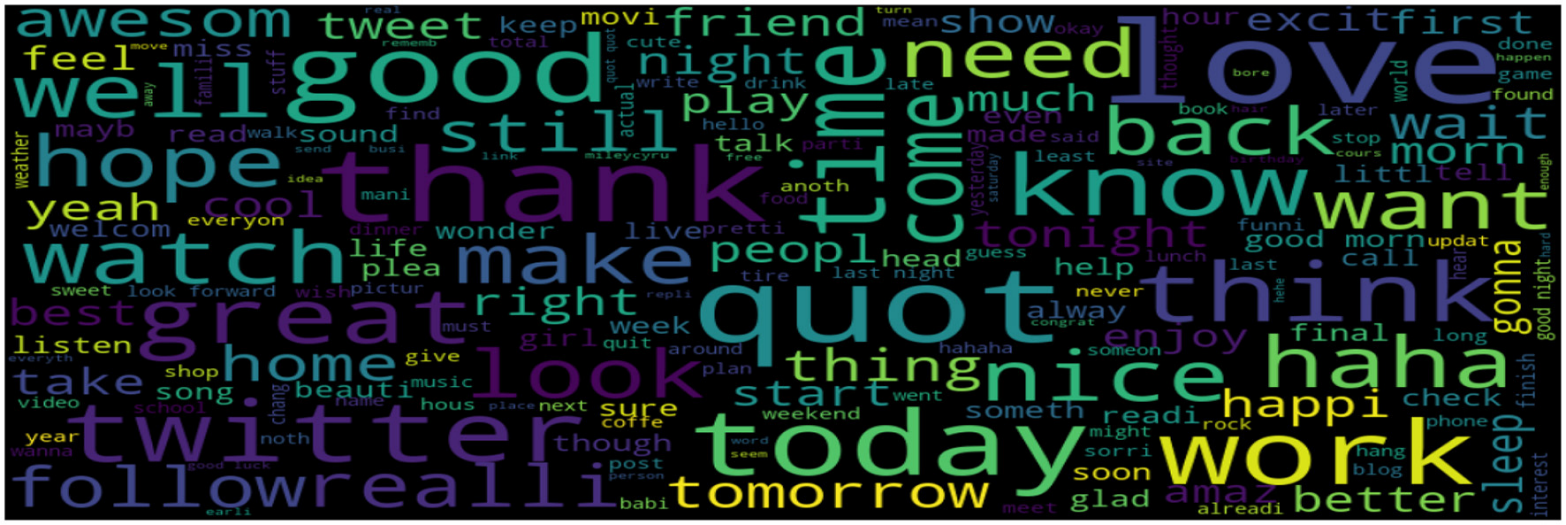
The most common positive words.

**Figure 8:**
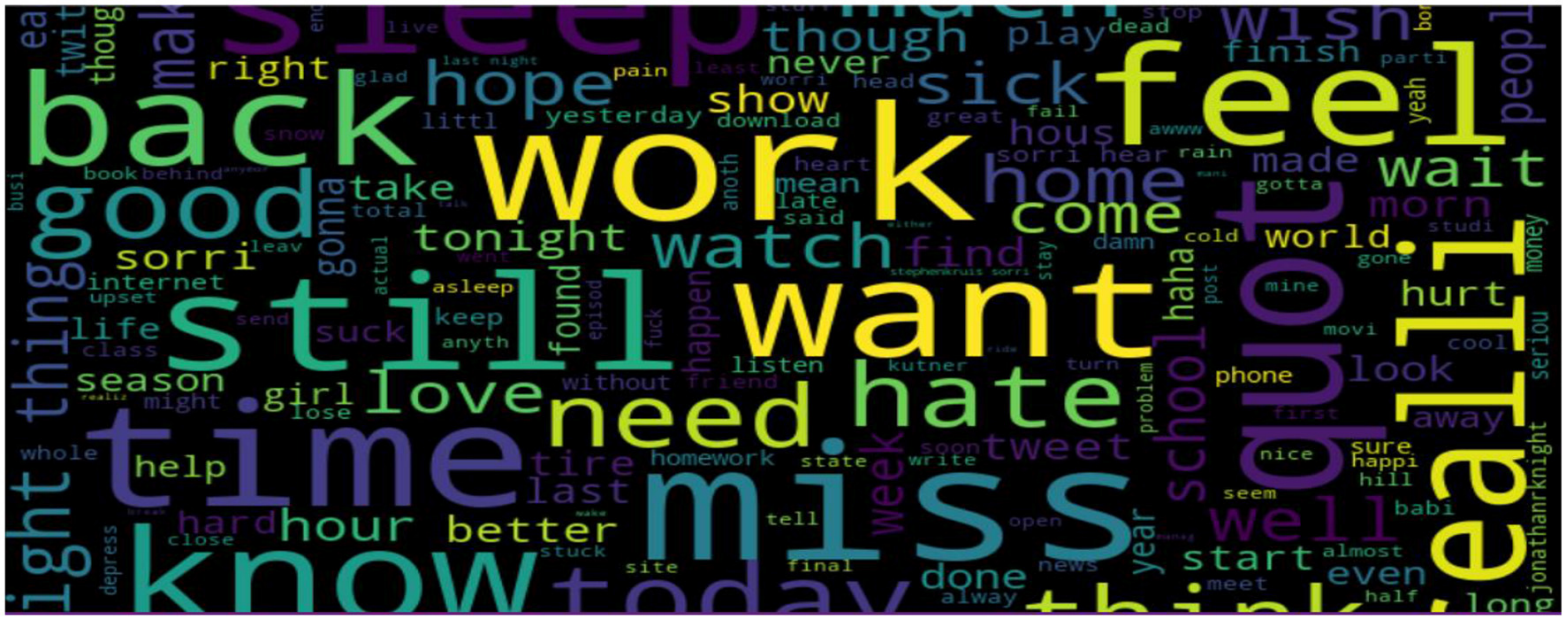
The most negative/depressed words.

The classification of a tweet's sentiment polarity is depicted in Figures 6–8. Word clouds were used to visualize the Tweets' Sentiment Polarity. Figure 6 depicts the most common words in the entire dataset, Figure 7 shows the most common positive words, and Figure 8 depicts the most negative/depressed words.

#### 3.1.5. Datasets

The datasets used for this study were a collection of Twitter datasets related to depression and sentiment analysis from the Kaggle website. The pre-processing stage of the study was a little difficult due to the different structures of the datasets as some datasets contained target values pre-set sentiments while others did not. Similar columns that were required were the tweets column and the id column.

**Figure 9:**
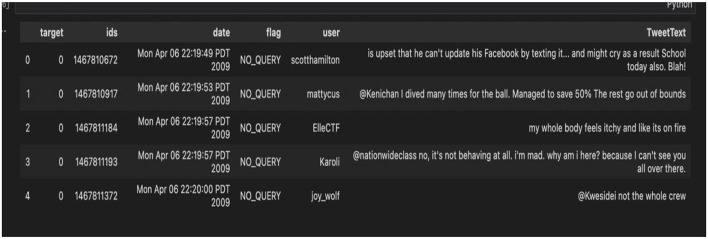
Complete dataset before selection of columns.

**Figure 10:**
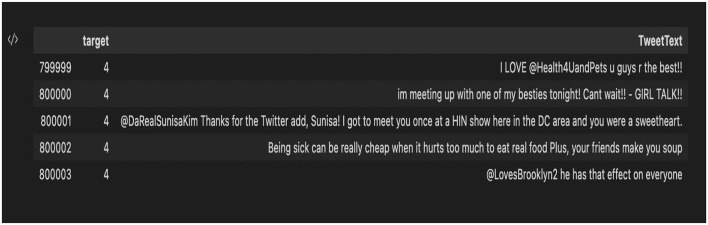
Dataset after column selection.

Out of the five datasets used, the “training.1600000. processed.noemoticon” dataset was the most useful. This dataset contained 1,599,999 rows × 6 columns. Of the six features, only two of the six features were concentrated on: the target column and the TextTweet. The Clean_TweetText column was then added that contained the cleaned tweets. Figure 9 shows the complete dataset before feature selection, and Figure 10 shows the dataset after feature selection was done.

## 4. Performance evaluation metrics

In this study, the evaluation of four machine learning models, namely XGB Classifier, Random Forest Classifier, Logistic Regression, and Support Vector Machine C-Support Vector Classification Model, was performed using the confusion matrix. The effectiveness of the models' predictions was assessed using metrics such as the accuracy score.

**Figure 11:**
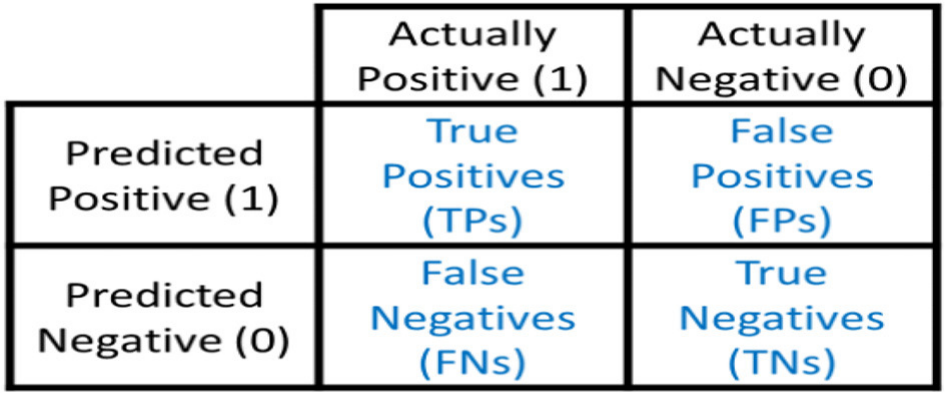
Confusion matrix (Draelos, 2019).

The confusion matrix shown in Figure 11 is organized into four categories:

1. True Positives (TP): Instances where the model correctly predicts tweets expressing depression sentiment.
2. True Negatives (TN): Instances where the model correctly predicts tweets not expressing depression sentiment.
3. False Positives (FP): Instances where the model incorrectly predicts tweets as expressing depression sentiment when they do not (a Type I error).
4. False Negatives (FN): Instances where the model incorrectly predicts tweets as not expressing depression sentiment when they do (a Type II error).

The confusion matrix allows us to calculate several evaluation metrics:

1. Accuracy: It measures the overall correctness of the model and is calculated as (TP + TN)/(TP + TN + FP + FN). The accuracy values for different classifiers are given for comparison. The accuracy metric is commonly used to evaluate the performance of a classification model.
2. Precision: It indicates the proportion of correctly predicted positive instances out of the total instances predicted as positive. Precision is calculated as TP/(TP + FP).
3. Recall (Sensitivity or True Positive Rate): It represents the proportion of correctly predicted positive instances out of the total actual positive instances. Recall is calculated as TP/(TP + FN).
4. F1 Score: It is the harmonic mean of precision and recall, providing a balance between the two metrics. The F1 score is calculated as 2 ^*^ (Precision ^*^ Recall)/(Precision + Recall).

## 5. Experiment and results

This section looks at the results presented by the individual models before discussing which of the models was best and how this was concluded. The comparison process and rating criteria were based on two factors. The accuracy of the model and the time the model took to execute. Our final study looked at comparing four models on the same dataset.

### 5.1. Machine learning classifier

The four models we looked at included python XGB Classifier, Random Forest Classifier, Logistic Regression, and Support Vector Machine C-Support Vector Classification Model. The four models are described in the Sections 5.1.1–5.1.4.

#### 5.1.1. XGB classifier

**Figure 12:**
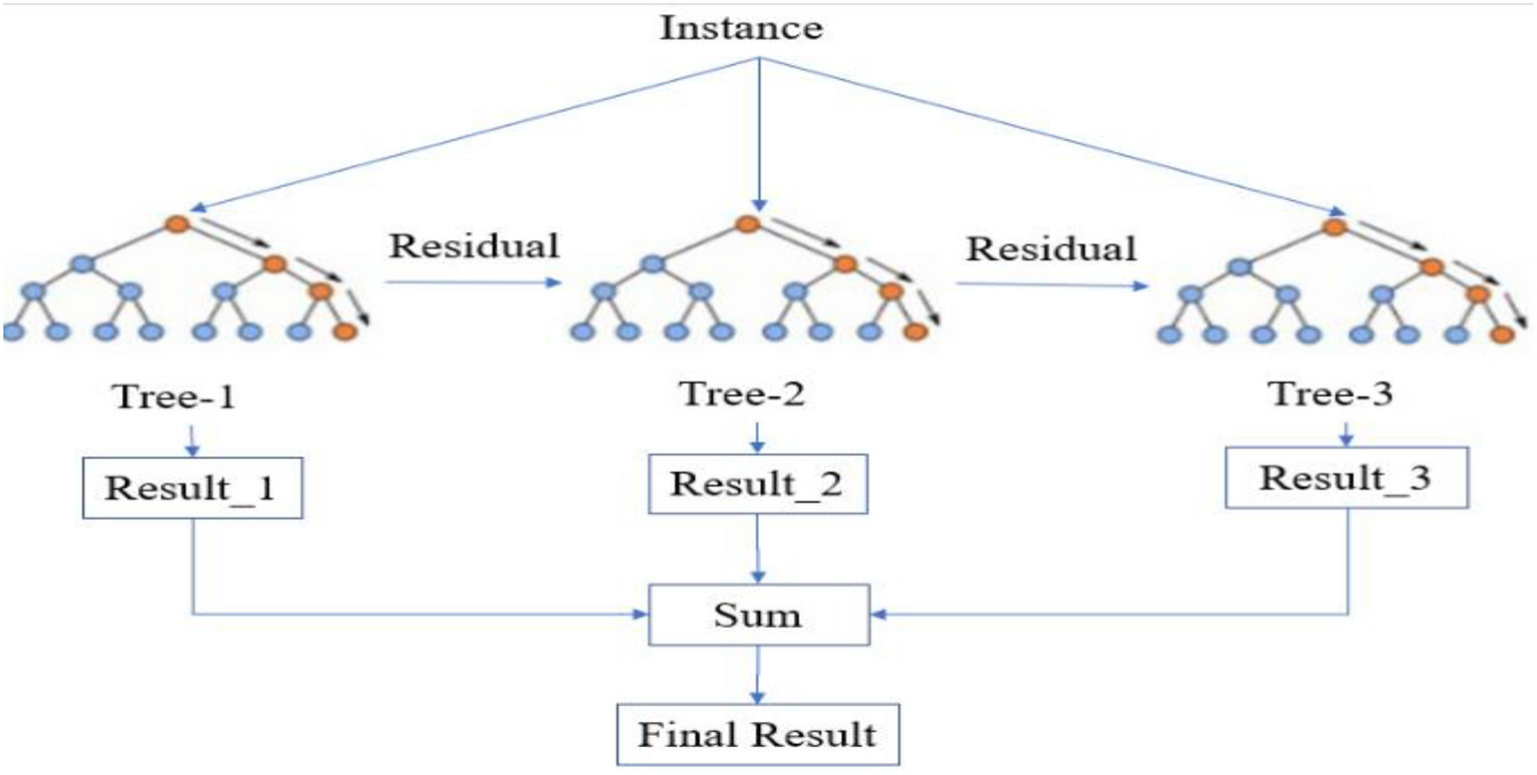
Simplified XGB classifier (Wang et al., 2020).

XGB Classifier is a machine learning model popular for its speed and accuracy, and it is widely used in different industries for solving classification problems. This model is primarily designed to solve classification problems by creating a set of decision trees iteratively, hence uses a decision tree ensemble method called Gradient Boosting. Moreover, in each iteration, the model identifies the instances that were not classified correctly in the previous iteration and focuses on them to improve the accuracy of the model. Figure 12 illustrates the functioning process of the model.

#### 5.1.2. Random forest

Random Forest model is an ensemble learning method that combines the predictions of multiple decision trees. It is commonly used for both classification and regression tasks in various domains. Random Forests are known for their ability to handle high-dimensional data, reduce overfitting, and provide robust predictions. It randomly selects subsets of features and training data to build each tree independently. The predictions of the individual trees are then aggregated to make the final prediction.

**Figure 13:**
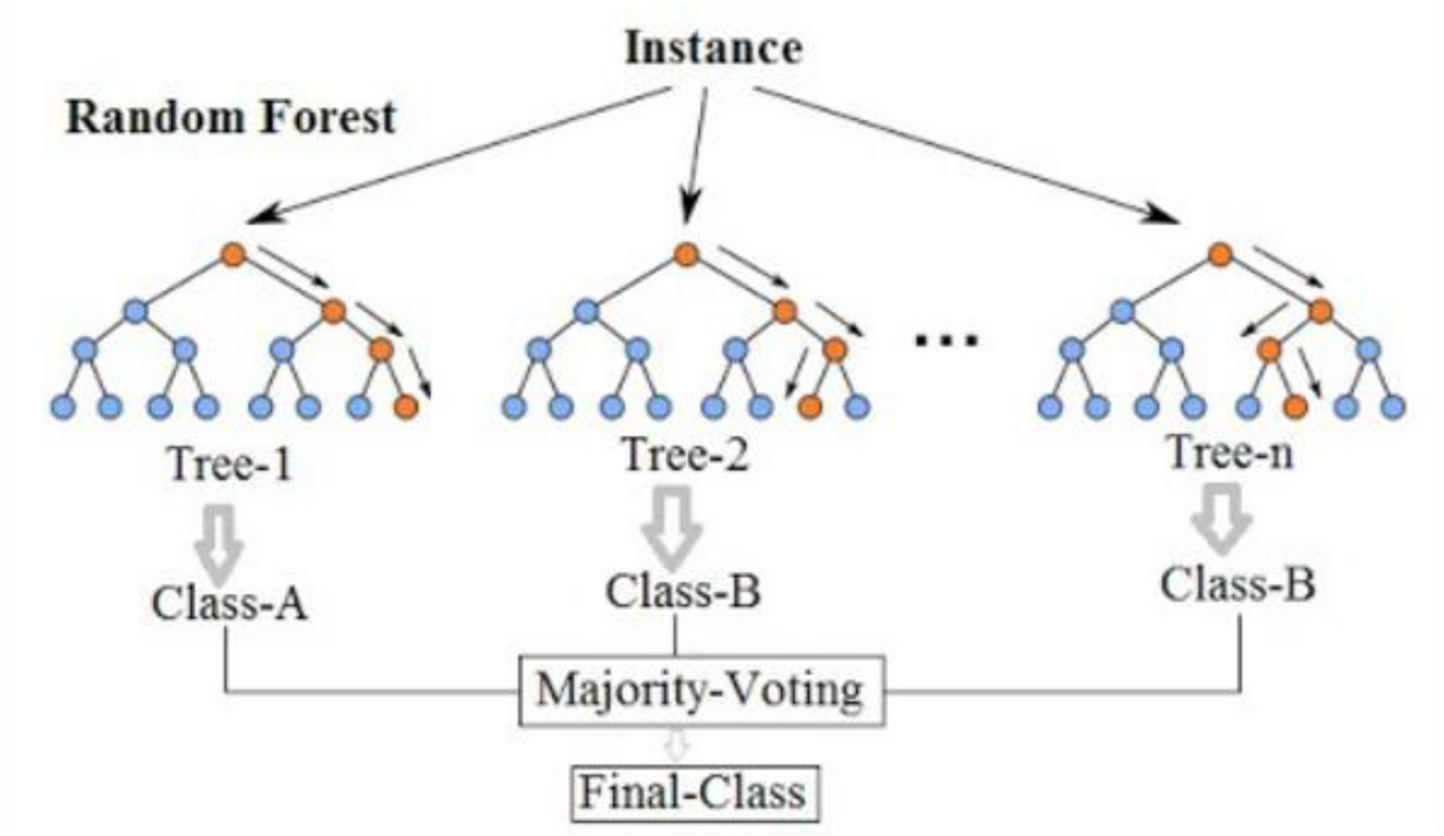
Simplified random forest model (Wikipedia, 2023).

Figure 13 shows how a random forest model works.

#### 5.1.3. Logistic regression

**Figure 14:**
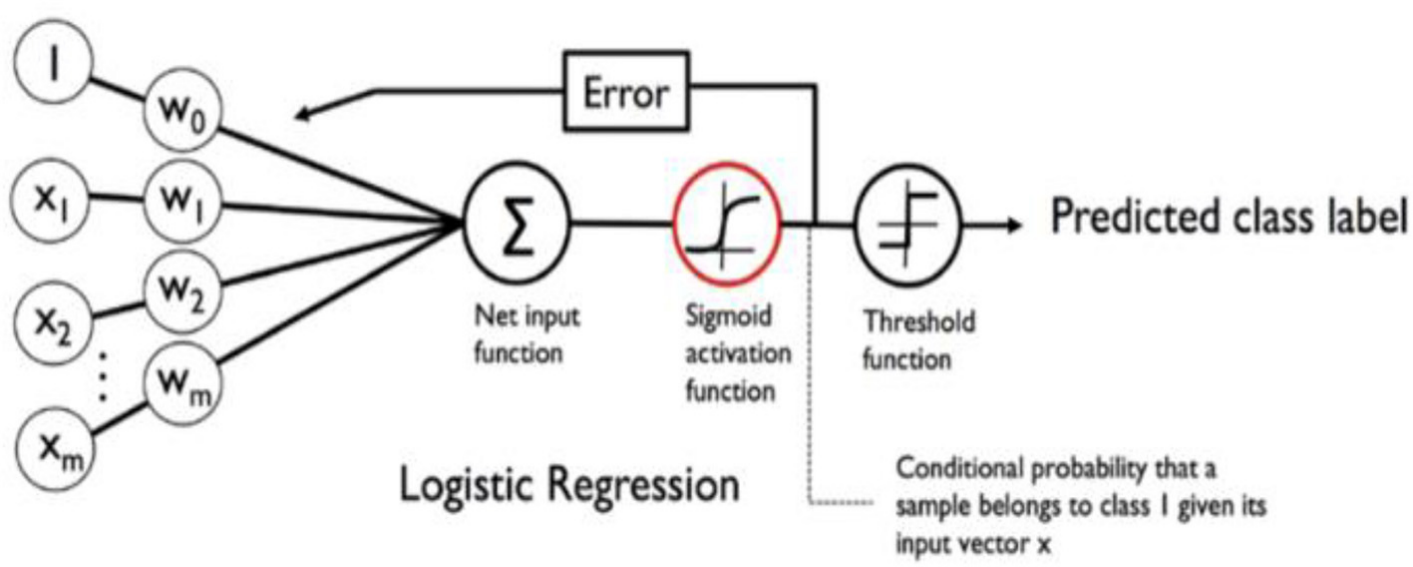
Logistic regression model (Torres et al., 2019).

Logistic regression is a machine learning algorithm specifically designed for predicting categorical dependent variables with binary outcomes, such as yes or no, true or false, or 0 or 1. It models the relationship between the input features and the probability of the binary outcome using a logistic or sigmoid function. By estimating coefficients through training, the algorithm maximizes the likelihood of the observed data. The predicted probabilities can be transformed into binary predictions using a threshold value. Logistic regression is favored for its simplicity and interpretability, although it assumes a linear relationship between the features and may have limitations in complex scenarios. The model's performance is commonly evaluated using metrics like accuracy and precision. See the depiction of the Logistic Regression model in Figure 14.

#### 5.1.4. Support vector machine

Support Vector Machines (SVM) are machine learning models that are versatile and used for classification and regression tasks. They aim to find an optimal decision boundary that maximizes the margin between classes. SVM utilizes the kernel trick to handle non-linear data, and support vectors are crucial in defining the decision boundary. The model is trained by optimizing the boundary and balancing regularization parameters. SVM is effective in handling high-dimensional data and complex decision boundaries.

**Figure 15:**
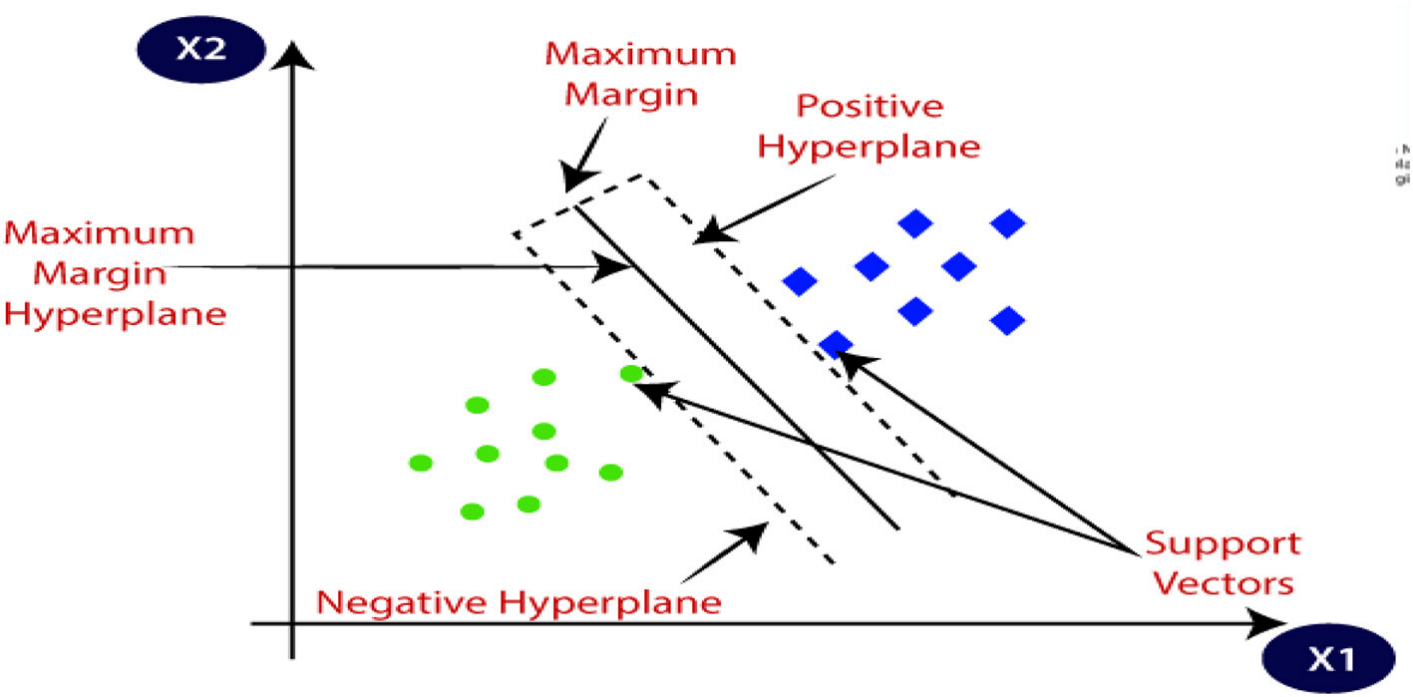
Support vector machine model (JavaTpoint, 2011–2021).

Figure 15 is a depiction of SVM.

### 5.2. Results obtained from the models

Table 1 showcases the results of different models, namely XGB Classifier, Random Forest, Logistic Regression, and SVM/SVC. The table presents their performance based on accuracy scores and computation time in seconds. The accuracy scores range from 95.2 to 96.3%, while the computation time varies significantly across the models, with values ranging from 0.29 to 1,072.32 s. These results provide insights into the models' predictive accuracy and computational efficiency, serving as a basis for further analysis and comparison.

**Table 1:** Model results.

**Rating criteria**	**XGB classifier**	**Random forest**	**Logistic regression**	**SVM**
Accuracy	96.1%	95.2%	96.3%	96.2%
Computation time (s)	6.75	1,072.32	0.29	29.92

The individual results of these models are presented in Table 1.

The results presented suggest that the SVM model and Logistic Regression model produced the most accurate results, with Logistic Regression slightly outperforming the SVM model, while the logistic regression model computed in the shortest amount of time. A more detailed analysis of the results suggests that the accuracy of the results was relatively similar for all four models. The lowest of the four models was the Random Forest Model with an accuracy of 95.2%, surprising as this is an Ensemble method, and it also had the longest computational time of 1,072.32 s. The second was the XGB Classifier with an accuracy of 96.1% and a computational time of 6.75 s. The third is the SVM Model with an accuracy of 96.2% and a computational time of 29.92 s and again, the fastest was the Logistic Regression model with an accuracy of 96.3% and a computational time of 0.29 s.

## 6. Discussion of results

The choice of model in the analysis process depends on the specific objectives of the study. In this case, the goal is to identify signs of depression in tweets. The effectiveness of the models can be evaluated based on two key factors: speed and accuracy. If the primary objective is to identify depression in tweets at a fast pace, models with faster computation times would be more suitable. These models may sacrifice some accuracy for speed. On the other hand, if accuracy is of utmost importance, models with higher accuracy rates should be prioritized, even if they have longer computation times. Considering the focus of this research on identifying depression signs in real-time as tweets come in, it is crucial to have a model that can classify them quickly and accurately. Therefore, it is necessary to analyze both the computational time and accuracy of the models to make an informed decision.

Based on the results shown in Table 1, the Logistic Regression model stands out as the most effective option. It achieved the highest accuracy rate of 96.3% while maintaining a relatively low computational time of 0.29 s. This combination of high accuracy and fast computation makes it a strong contender for solving the depression identification problem in real-time tweet analysis. Looking at this, this paper can clearly state the Logistic Regression model emerges as the most suitable choice. It balances both accuracy and computational time, making it an effective tool for identifying signs of depression in tweets.

### 6.1. Comparison with existing studies

When comparing the results with previous studies, several insights emerge. Previous studies, however, suggest that ensemble methods should be more effective in sentiment analysis. In the comparison of the accuracy scores presented in Table 2, the random forest classifier's results are quite alarming, considering the higher expectations for ensemble methods. Jain et al. (2022) conducted a similar study and confirmed the effectiveness of the SVM classifier, which outperformed logistic regression and random forest in three out of the represented categories. This aligns with the findings of Jianqiang and Xiaolin (2017), who also highlighted the superior performance of SVM compared to logistic regression and random forest in their study.

**Table 2:** Presents a comparison of the accuracy scores of the four models with previous studies, highlighting their performance.

**ML models**	**Jain et al., 2022**	**Aliman et al., 2022**	**Dave, 2023**	**Sujithra et al., 2023**	**Aljabri et al., 2022**	**This study**
Logistic regression	79% (highest)	81% (highest)	83.62%	74.78%	N/A	96.3% (highest)
SVM/SVC	77.12%	69%	86.95%	N/A	88%	96.2%
XGB classifier	N/A	N/A	86.76%	74.22%	90% (highest)	96.1%
Random forest	77.298%	N/A	88.38% (highest)	75.12% (highest)	78%	95.2%

Interestingly, Dave (2023) reported a relatively higher accuracy score for logistic regression compared to other models, reaching 83.62%. In addition, this study presents an even higher accuracy score for logistic regression, at 96.3%, indicating its effectiveness in accurately predicting the presence of depression sentiment in tweets. Hence the results obtained in this study indicate that logistic regression emerged as the most suitable model for the paper.

The SVM model, although not consistently outperforming the other models across previous studies, still demonstrates competitive accuracy scores. For instance, in the study by Aljabri et al. (2022), SVM achieved an accuracy of 88%. Similarly, in the current study, SVM/SVC performed well with an accuracy score of 96.2%.

The XGB Classifier achieved an accuracy of 96.1% in this study, indicating its strong performance in detecting depression sentiment in tweets. Compared to other models in the study, the XGB Classifier had the third highest accuracy score. Additionally, one previous study by Aljabri et al. (2022) reported a high accuracy of 90% for the XGB Classifier, further highlighting its effectiveness. The XGB Classifier's ability to capture complex patterns and interactions in the data likely contributed to its successful performance. Overall, the XGB Classifier shows promise as a reliable model for depression detection in tweets.

On the other hand, the random forest model presents mixed results. While it achieved the highest accuracy score in the study by Aliman et al. (2022) at 88.38%, it obtained a relatively lower score in the current study, with 95.2%. These variations could be attributed to different datasets or other factors.

Overall, considering the consistently high accuracy scores and the specific requirements of the paper, logistic regression emerged as the best model choice. However, the inclusion of other models such as SVM and XGB Classifier allows for a comprehensive comparison and exploration of their performance in sentiment analysis.

## 7. Conclusion

The paper aimed to identify depression using user tweets more reliably early. As a result, this research proposed a tool based on four classifiers, NLP, and sentiment analysis techniques to improve performance in the early detection of depression. A series of experiments were carried out to evaluate the accuracy and efficacy of the four classification models (XGBClassifier, Random Forest, Logistic Regression, and SVM) that were used on the four datasets combined as one. The results show that the Logistic Regression and SVM models were the most accurate, with Logistic Regression outperforming the SVM model slightly. However, the Logistic regression model was the fastest in terms of computational time of the depressive tweets. Future research should investigate ways to reduce computational time, while also improving model accuracy during the predictive process. Furthermore, in the extension of this work, we are interested in testing the model on new datasets to detect depression.

## Data availability statement

The original contributions presented in the study are included in the article/supplementary material, further inquiries can be directed to the corresponding author.

## Author contributions

IO, SD, and OC: study conception and design, analysis and interpretation of results, and draft manuscript preparation. SD and OC: data collection. All authors reviewed the results and approved the final version of the manuscript.

## References

[B1] Aliman G. Nivera T. Olazo J. Ramos D. J. Sanchez C. Amado T. Valenzuela I. C. (2022). Sentiment analysis using logistic regression. J. Comp. Innovat. Eng. Appl. 35–40.

[B2] Aljabri M. Aljameel S. S. Khan I. U. Aslam N. Charouf S. M. Alzahrani N. (2022). Machine learning model for sentiment analysis of COVID-19 tweets. Int. J. Adv. Sci. Eng. Inf. Technol. 12, 1206–1214. 10.18517/ijaseit.12.3.14724

[B3] AlSagri H. S. Ykhlef M. (2020). Machine learning-based approach for depression detection in twitter using content and activity features. IEICE Transact. Inf. Syst. 103, 1825–1832. 10.1587/transinf.2020EDP7023

[B4] Babu N. V. Kanaga E. G. M. (2022). Sentiment analysis in social media data for depression detection using artificial intelligence: a review. SN Comp. Sci. 3, 1–20. 10.1007/s42979-021-00958-1 34816124 PMC8603338

[B5] Dave G. H. (2023). Leveraging big data for early detection of depression: developing a machine learning model using tweets. Vidhyayana Int. Multidiscipl. Peer Rev. Eur J. 8, 777–784. Available online at: https://vidhyayanaejournal.org/journal/article/view/784

[B6] Draelos R. (2019). GLASS BOX - Machine Learning and Medicine. Available online at: https://glassboxmedicine.com/2019/02/17/measuring-performance-the-confusion-matrix/ (accessed October 30, 2022).

[B7] Gaikwad S. Khairnar U. Deshpande A. (2014). Text mining process: Techniques and tools. Int. J. Adv. Res. Comput. Engg. Technol. 3, 413–418. Available online at: http://csjournals.com/IJITKM/PDF%203-1/86.pdf

[B8] Jagadishwari V. Indulekha A. Kiran R.aghu, Harshini, P. (2021). Sentiment analysis of social media text-emoticon post with machine learning models contribution title. J. Phys. 2070, 012079. 10.1088/1742-6596/2070/1/012079

[B9] Jain P. Srinivas K. Vichare A. (2022). Depression and suicide analysis using machine learning and NLP. J. Phys. 2161, 012034. 10.1088/1742-6596/2161/1/012034

[B10] JavaTpoint (2011–2021). JavaTpoint. Available online at: https://www.javatpoint.com/machine-learning-support-vector-machine-algorithm (accessed June 14, 2023).

[B11] Jianqiang Z. Xiaolin G. (2017). Comparison research on text pre-processing methods on twitter sentiment analysis. IEEE Access 5, 2870–2879. 10.1109/ACCESS.2017.2672677

[B12] Kumar P. Garg S. Garg A. (2020). Assessment of anxiety, depression and stress using learning models. Proced. Comput. Sci. 171, 1989–1998. 10.1016/j.procs.2020.04.21335735574

[B13] Lui C. (2020). Social media and public opinion during COVID-19 pandemic: A cross-country analysis. Comput. Hum. Behav. 110, 106380. 10.1016/j.chb.2020.106380 32292239 PMC7151317

[B14] Mun B. Kim H. (2021). Influence of false self-presentation on mental health and deleting behavior on instagram: the mediating role of perceived popularity. Front. Psychol. 12, 660484. 10.3389/fpsyg.2021.660484 33912119 PMC8071929

[B15] Orabi A. Buddhitha P. Orabi M. Inkpen D. (2018). “Deep learning for depression detection of twitter users,” in Proceedings of the Fifth Workshop on Computational Linguistics and Clinical Psychology: From Keyboard to Clinic (New Orleans, LA: Association for Computational Linguistics), 88–97.

[B16] Orsolini L. Pompili S. Salvi V. Volpe U. (2021). A systematic review on telemental health in youth mental health: Focus on anxiety, depression and obsessive-compulsive disorder (Medicina: MDPI). 57, 793. 10.3390/medicina57080793 34440999 PMC8398756

[B17] Priya A. Garg S. Tigga N. (2020). Predicting anxiety, depression, and stress in modern life using machine learning algorithms. Proced. Comput. Sci. 167, 1258–1267. 10.1016/j.procs.2020.03.442

[B18] Ranganathan J. Tzacheva A. (2019). Emotion mining in social media data. Proc. Comp. Sci. 159, 58–66. 10.1016/j.procs.2019.09.160

[B19] Ricard B. Marsch L. Crosier B. Hassanpour S. (2018). Exploring the utility of community-generated social media content for detecting depression: an analytical study on instagram. J. Med. Int. Res. 20, e118. 10.2196/11817 30522991 PMC6302231

[B20] Samsari N. S. Mohamad M. Selamati A. (2022). Sentiment analysis on students' stress and depression due to online distance learning during the COVID-19 pandemic. Math. Sci. Inf. J. 3, 66–74. 10.24191/mij.v3i1.18273

[B21] Seabrook E. Kern M. L. Fulcher B. D. Rickard N. S. (2018). Predicting depression from language-based emotion dynamics: a longitudinal analysis of Facebook and Twitter status updates. J. Med. Int. Res. 20, e168. 10.2196/jmir.9267 29739736 PMC5964306

[B22] Seney M. Zhiguang H. Cahill K. L. F. Puralewski R. Zhang J.. (2018). Opposite molecular signatures of depression in men and women. Biol. Psychiatry. 84, 8–27. 10.1016/j.biopsych.2018.01.017 29548746 PMC6014892

[B23] Shukla V. S. Vala J. (2016). A survey on image mining, its techniques, and application. Int. J. Comp. Appl. 133, 12–15. 10.5120/ijca2016907978

[B24] Smith R. Anderson M. (2018). Instagram photos reveal predictive markers of depression. EPJ Data Sci. 7, 15. 10.1140/epjds/s13688-018-0140-6

[B25] Sood A. Hooda M. Dhir S. Bhatia M. (2018). An Initiative To Identify Depression Using Sentiment Analysis: A Machine Learning Approach. Indian J. Sci. Technol. 11, 1–6. 10.17485/ijst/2018/v11i4/119594

[B26] Sujithra M. Rathika J. Velvadivu P. Marimuthu M. (2023). An intellectual decision system for classification of mental health illness on social media using computational intelligence approach. J. Ubiquit. Comp. Commun. Technol. 5, 23–35. 10.36548/jucct.2023.1.002

[B27] The University of Queensland (2022). University of Queensland. Available online at: https://qbi.uq.edu.au/brain/brain-diseases/depression (accessed August 04, 2022).

[B28] Tiwari A. Mishra A. Rath S. K. (2021). “Emotion mining in social media data,” in Intelligent Computing Techniques for Cyber Security (Singapore: Springer), 227–238.

[B29] Torres R. Ohashi O. Pessin G. (2019). A machine-learning approach to distinguish passengers and drivers reading while driving. Sensors (Basel). 19:3174. 10.3390/s19143174 31330929 PMC6679284

[B30] Wang W. Chakraborty G. Chakraborty B. (2020). Predicting the Risk of Chronic Kidney Disease (CKD) Using Machine Learning Algorithm. ResearchGate. Available online at: https://www.researchgate.net/figure/Simplified-structure-of-XGBoost_fig2_348025909 (accessed June 14, 2023).

[B31] Wikipedia (2023). Wikipedia Organisation. Available online at: https://en.wikipedia.org/wiki/Random_forest (accessed June 14, 2023).

[B32] Zulfiker M. Kabir N. Biswas A. Nazneen T. Uddin M. (2021). An In-Depth Analysis of Machine Learning Approaches to Predict Depression. Elsevier, 38–50.

